# Organisational support for employees with dyslexia: An explorative study in South Africa

**DOI:** 10.4102/ajod.v14i0.1597

**Published:** 2025-05-16

**Authors:** Carmen Venter, Lizelle Rossouw

**Affiliations:** 1 WorkWell Research Unit, Faculty of Economic and Management Sciences, North-West University, Potchefstroom, South Africa

**Keywords:** dyslexia, adult with dyslexia, employee with dyslexia, organisational support, South African organisations, specific learning disability

## Abstract

**Background:**

Dyslexia is a neurodevelopmental disorder that affects reading, writing and spelling. While it is often identified and accommodated in educational settings, employees with dyslexia (EWD) may still face challenges in the workplace as they continue to struggle with this disability throughout their adult lives. While dyslexia can pose challenges for adults in the workplace, accommodations and support measures are available to help mitigate these difficulties.

**Objectives:**

This study explored the nature of organisational support provided to EWD within South African organisations.

**Method:**

This qualitative research study adopted a constructivist paradigm and applied a qualitative descriptive research strategy. The research approach involved conducting 15 (*N* = 15) semi-structured virtual interviews with EWD.

**Results:**

Employees with dyslexia identified challenges related to dyslexia. Some reported efficient organisational support, while others felt it was lacking. Many used adaptive strategies to cope with daily difficulties. Recommendations were made to improve support for EWD.

**Conclusion:**

Employees with dyslexia’s experiences can inform the development of inclusive policies and practices supporting these individuals. Moreover, EWD highlighted the importance of raising awareness and promoting a culture of inclusivity and support for dyslexia within South African organisations.

**Contribution:**

The study contributes to the literature on dyslexia and the workforce regarding organisational support within a South African context and has captured the need to encourage heightened awareness, empathy and equitable practices within organisations.

## Introduction

Dyslexia, classified as a specific learning disability (SLD) by the American Psychiatric Association (2022), poses a global risk to approximately 700 million individuals, potentially leading to lifelong challenges such as the inability to read or write as well as social exclusion (Geertseman et al. 2022). The available literature on the impact of dyslexia in the workplace is somewhat limited, especially on office jobs and the cognitive aspect related to performing daily duties in this type of environment (Smith-Spark, Gordon & Jansari 2022). A more detailed definition of dyslexia is provided by The International Dyslexia Association (2022), which states that:

Dyslexia is a specific learning disability that is neurobiological in origin. It is characterised by difficulties with accurate and fluent word recognition and poor spelling and decoding abilities. Secondary consequences may include problems in reading comprehension and reduced reading experience that can impede the growth of vocabulary and background knowledge. (p. 1)

Organisational tasks such as understanding written reports, producing written work quickly, note-taking, logically structuring a piece of work, over-checking and double-checking work and copying or recalling numbers are severely affected in employees with dyslexia (EWD) because of difficulties with reading, writing and spelling, which are inherent with dyslexia (ed. Moody 2015). In essence, a dyslexic individual might experience difficulties in processing information (Bartlett, Moody & Kindersley 2010; Del Tufo & Earle 2020). In addition, as technology and digital communication advance, workplace success is increasingly contingent upon one’s literacy skills, leaving those with dyslexia disadvantaged in the professional sphere (Wissell et al. 2022a). Considering the challenges EWD face concerning reading, spelling and writing, they face an elevated likelihood of being unable to meet the requirements or deadlines of their workplace, even if they possess the necessary training and qualifications (Wissell et al. 2022b). In addition, being dyslexic can lead to significant frustration, especially given that the skills it impacts are foundational within a professional setting (Sanderson 2011).

In addition to the challenges these employees face, there is also an extreme lack of awareness within the global world, especially in the world of work, where there are common misconceptions, leading to misjudgements and misunderstandings of what a dyslexia diagnosis comprehends (Yong, Ng & Nakayama 2019). Those employed, despite being dyslexic, report feeling ashamed and embarrassed about their difficulties with reading and writing, which frequently leads to lower self-esteem and confidence in the organisation (Snowling 2019). One significant challenge arises because of lack of information; without a proper understanding of dyslexia, employees might hesitate to seek the necessary support, and organisations could remain unaware of available resources, once again underscoring the importance of promoting dyslexia awareness and available support or accommodations in the workplace (Beetham & Okhai 2017; Sanderson 2011).

Wissell et al. (2022b) stated that in the absence of sufficient support or accommodations in the workplace, EWD may struggle to meet the requirements laid down in the workplace. Failure to obtain organisational support or accommodations can lead to lower performance levels; thus, the dyslexic employee may experience lower levels of well-being (Beetham & Okhai 2017). Research suggests that because of the lack of comprehensive training for employers and managers on effectively collaborating with and supporting EWD, dyslexia remains unrecognised. Hence, the onus is placed on EWD to disclose their disability and advocate for the necessary support (O’Dwyer & Thorpe 2013; Thorpe & Burns 2016; Wissell et al. 2022b). Unfortunately, people living with disabilities are disproportionately more likely to struggle to find employment than those without disabilities (Wissell et al. 2022a).

De Beer et al. (2014) point out that there have been improvements in educational provisions for children and adolescents with dyslexia, including assistive technology, time extensions and adaptations such as recorded textbooks and larger fonts; these enhancements have not been as readily evident within the workplace settings. This becomes especially noteworthy as an increasing number of well-educated young adults managing their dyslexia through educational support and adaptions step into comparatively less equipped organisations to provide similar accommodations and assistance, highlighting a noticeable gap between educational institutions and the workplace (De Beer et al. 2014). Choudhury (2023) stated that the most significant challenges with dyslexia are inadequate assistance in the workplace, limited consideration from higher-level management roles and a lack of concern for different individuals.

The Code of Good Practice on the Employment of Persons with Disabilities highlights that providing equal opportunities and reasonable accommodations or support to individuals with disabilities empowers them to contribute their valuable skills to the organisation (Department of Labour 2015). Similarly, South Africa’s *Employment Equity Act 55 of 1998*, detailed in the Government Gazette (2015), is dedicated to eradicating unfair discrimination and ensuring equitable representation of people with disabilities in the workplace, explicitly emphasising implementing reasonable accommodations (Department of Labour 2015). Willingness on the part of employers to implement supportive measures for employees with disabilities, such as dyslexia, can substantially enhance an organisation’s productivity and overall staff morale (Sanderson 2011). However, employers face challenges in supporting EWD. There is no standardised blueprint for supporting EWD, as there are several types and combinations of dyslexia (Kirby & Gibbon 2018). Thus, to truly support a dyslexic employee, one needs to develop an individual-specific support programme (Kirby & Gibbon 2018). Given this individualised nature of support and accommodation requirements for EWD within organisations, it is crucial to determine effective strategies for meeting their unique needs and enhancing their perception of organisational support.

The perceived organisational support (POS) model stems from the organisational support theory (OST) and posits that employees will develop their own general beliefs or perceptions concerning how much an organisation values their contribution to the workplace as well as cares about their well-being (Kurtessis et al. 2017). According to OST, POS is mainly concerned with the employees’ perceptions of the motivations behind the treatment received from the organisation, whether good or bad (Kurtessis et al. 2017). Perception of organisational support initiates a process of change, as employees will experience a feeling of obligation to help the organisation reach goals; they will, therefore, expect greater rewards and benefits (Kurtessis et al. 2017). Favourable employee treatment within an organisation directly influences their POS (Maan et al. 2020; Rhoades & Eisenberger 2002). Oubibi et al. (2022) also confirmed a positive relationship between POS and job satisfaction. Research also indicates that when employees experience high levels of POS, it could lead to promising outcomes such as high levels of organisational commitment, higher levels of performance and productivity and positive emotions (Maan et al. 2020; Yu & Frenkel 2013). It is evident that when an employee experiences high levels of POS, the organisation will benefit greatly. The POS model was chosen as the basis of this research study to emphasise the importance of providing employees, especially employees with disability, with the necessary support. Literature highlights the benefits the organisation will experience when doing so.

This study addresses a critical gap in the research on dyslexia. Existing literature concentrates on children with dyslexia (Cheng et al. 2022; Li & Bi 2022), students with dyslexia (Donato et al. 2022; Jacobs et al. 2022), dyslexia and the stigma thereof (Deacon, Macdonald & Donaghue 2022) and the impairments or difficulties associated with developmental dyslexia (Liu et al. 2022; Protopapa & Smith-Spark 2022). The perspective of EWD and organisational support remains unexplored (Reid, Came & Price 2008; Young & Browning 2004). This study aims to investigate the experiences of EWD in South African organisations, specifically, whether they receive support, the nature of that support and its perceived effectiveness. By focusing on workplace support for dyslexic adults, this research seeks to bridge the current knowledge gap and inform best practices for employers.

## Research methods and design

### Study design

This research study adopted the constructivist paradigm as its guiding framework. This paradigm was chosen as it seeks to explore people’s subjective meanings and experiences in their natural settings through qualitative research methods (Crotty 2016). By adopting the constructivist paradigm, this research study recognised the importance of acknowledging EWD’s diverse perspectives and subjective interpretations.

This research also employed a qualitative descriptive approach. Sandelowski (2000:336) defines a descriptive qualitative design as a ‘comprehensive summary of an event in the everyday terms of those events’. Therefore, this strategy was chosen as it assists the researcher in describing dyslexia and its organisational support within South African organisations. A qualitative descriptive research strategy was the most suitable approach for this research study as it acknowledges the fact that the experience of dyslexia is subject to personal interpretation and, thus, each participant may encounter it differently (Doyle et al. 2020). Furthermore, it enabled the researchers to present their findings in a manner that closely ties to the terminology used in the initial research questions (Doyle et al. 2020).

### Setting

This research study did not occur in one particular sector or industry, nor did it explicitly focus on one type of occupation. The research study did, however, require the participants to be diagnosed with dyslexia and employed within a South African organisation.

### Study population

A study’s population refers to the complete set of individuals, objects or events that share a common characteristic and are the focus of the study (Kivunja & Kuyini 2023). This study’s population included EWD. Dyslexia is categorised as a SLD, which is believed to be a neurodevelopment condition characterised by difficulty producing, comprehending and understanding written language (Castillo & Gilger 2018).

A combination of convenience and maximum variation sampling was chosen as sampling techniques for this study. Convenience sampling allows researchers to select participants who are easily accessible (Ary et al. 2019). Maximum variation sampling is a technique that explicitly selects participants who reflect a variety of traits or viewpoints (Patton 2014). The researcher gathered data from employees from various organisations and industries throughout South Africa to gain a diverse viewpoint of organisational support for EWD. These sampling methods were chosen as the researcher aimed to capture a variety of perspectives and experiences of the EWD. To be included in the study, the following applied:

- Employees diagnosed with dyslexia
- Individuals employed in a South African organisation for at least 6 months
- Employees fluent in Afrikaans or English
- Employees comfortable with computers
- Employees willingness to voluntarily participate in this study

The exclusion criteria for this research study included individuals employed less than 6 months in their current organisation and individuals who were only officially diagnosed with dyslexia in the past 6 months.

The study aimed for 20 participants but reached data saturation after 12 interviews. Three more interviews were conducted to ensure quality data, resulting in a final sample size of 15 (*N* = 15). The sample size was guided by data saturation and the criteria laid down by Morse (2020) when collecting quality data. Data were collected until the phenomenon under study could be comprehensively described.

The study participants were primarily middle-aged (42–52 years old, 40%), with a significant portion being young adults (20–30 years old, 33.3%). Women were slightly more represented (53.3%) than men (46.7%). Afrikaans was the primary language (86.7%), with very few speaking English as their first language (13.3%). The majority of participants (60%) had a diagnosis of dysphoneidesia, while dysnemkinphoneidesia was the second most common (26.7%).

After approval for the study from the relevant committees was gained, the researcher contacted the Stark Griffin Dyslexia Academy (SGDA) to recruit participants. In 2019, SGDA’s assessment tool received official recognition from the Health Professions Council of South Africa (HPCSA) for its ability to diagnose and sub-categorise dyslexia. Stark griffin dyslexia academy incorporates an optional invitation to participate in future dyslexia research studies within their assessment consent form. This upfront disclosure allows individuals to choose involvement at the outset. Those interested in being contacted for future research provide their email addresses during the assessment process. The researcher recruited potential participants through a list provided by the academy. This ensured that all participants had a confirmed diagnosis of dyslexia.

### Data collection

To gather in-depth information, the researcher conducted interviews using a semi-structured format. Because of the participants’ geographical distribution across South Africa, virtual video conferencing software was chosen as the most suitable method. Recognising that all participants have dyslexia, the option to choose between Zoom and Microsoft Teams provided flexibility in their preferred platform. Virtual interviews were conducted in either Afrikaans or English to accommodate participant preferences. Following data collection, a certified language translator transcribed all interviews into Microsoft Word documents. The interview questions that were posed included:

- What challenges do you experience as a dyslexic employee, if any? Please elaborate on your answer.
- How does your organisation provide you, as a dyslexic employee, with the necessary support to adequately do your job?
- How does your organisation provide you, as a dyslexic employee, with the necessary resources (or aids) to adequately do your job?
- Do you experience the organisational support you have mentioned to be adequate? Please provide reasons for your answer.
- What type of organisational support would help you to do your job effectively and efficiently?
- Is there anything else you would like to share with me?

### Data analysis

For this research, a conventional approach to content analysis was chosen. Conventional content analysis is a data analysis method that aims to describe and report on a phenomenon when existing literature is limited (Hsieh & Shannon 2005). Using this type of content analysis, the researcher developed categories to organise the collected data. Kleinheksel et al. (2020) identified four steps involved in qualitative content analysis, which are:

- **Identify units of meaning:** During this step, the researcher identified words, a sentence or a statement representing an idea or concept in the data captured.
- **Label equivalent units with a code:** After the units of meaning were identified, each unit was labelled with a code, between one to three words, which described that specific unit.
- **Group similar codes into a category:** After labelling the units with a code, the codes somehow related to one another (in content or context) were organised. In the case of many codes, the researcher grouped the codes into subcategories.
- **Describe related categories with a theme:** Related categories, fitted together and represented an underlying meaning, were then grouped and organised jointly and labelled with a theme. The purpose of themes was to ultimately describe behaviours and experiences in the various categories or subcategories.

### Trustworthiness

To guarantee trustworthiness, the researcher followed the criterion set out by Anney (2014), Bryman (2016) and Guba and Lincoln (1994). This criterion refers to dependability, credibility, transferability and confirmability and was followed throughout this research study, thus ensuring the data and findings’ trustworthiness.

#### Credibility

Credibility guarantees the honest and timely interpretation and analysis of the research study’s findings, ensuring that the data truthfully and honestly represent the participants’ initial responses (Anney 2014). The researcher ensured credibility by involving an accredited professional translator to assist with the transcriptions of the virtual interviews. The researcher also ensured that the interviews were transcribed correctly by correlating the interviews with the written interview data. In addition, the researcher also utilised a co-coder to ensure that the data captured were interpreted correctly. The co-coder is an Industrial Psychologist knowledgeable in qualitative research and analysis. The co-coder employed the exact steps of data analysis as the researcher did.

#### Transferability

Transferability is a term used to describe how beneficial the research study’s results or conclusions are to people in other contexts or how relevant they are to different people’s circumstances (Connelly 2016). The researcher enhanced transferability in this study by giving extensive and thorough descriptions of the research background, location and participants and being open and honest about the data analysis.

#### Dependability

Dependability refers to the degree to which the reader can be assured that the data reported are supported by a research study (Anney 2014). According to Connelly (2016), dependability encompasses the stability of the data over time and the conditions under which the study was conducted. The researcher ensured the dependability of this research study by keeping process logs. The process logs are the researcher’s notes regarding all activities and decisions made during the research study.

#### Conformability

Confirmability refers to the extent to which other researchers can verify or corroborate the findings of a research study (Anney 2014). Furthermore, Connelly (2016) states that confirmability pertains to the consistency of results and their potential for replication. Overall, confirmability aids in ensuring that the data of the research study are accurate, concrete, verified and free from imaginative elements. It was a priority for the researcher to report on the responses and opinions of the participants in an honest, objective and accurate manner, thus ensuring that the data accurately represent the participant’s responses. The researcher prioritised remaining objective and neutral throughout the research study to reduce or eliminate the effect of potential investigator bias.

### Ethical considerations

This research received ethical approval from the Economic and Management Sciences Research Ethics Committee of the North-West University (ethics number: NWU-01839-22-A4). Ethical principles such as informed consent, voluntary participation, withdrawal rights, confidentiality and anonymity were followed.

## Results

**FIGURE 1:**
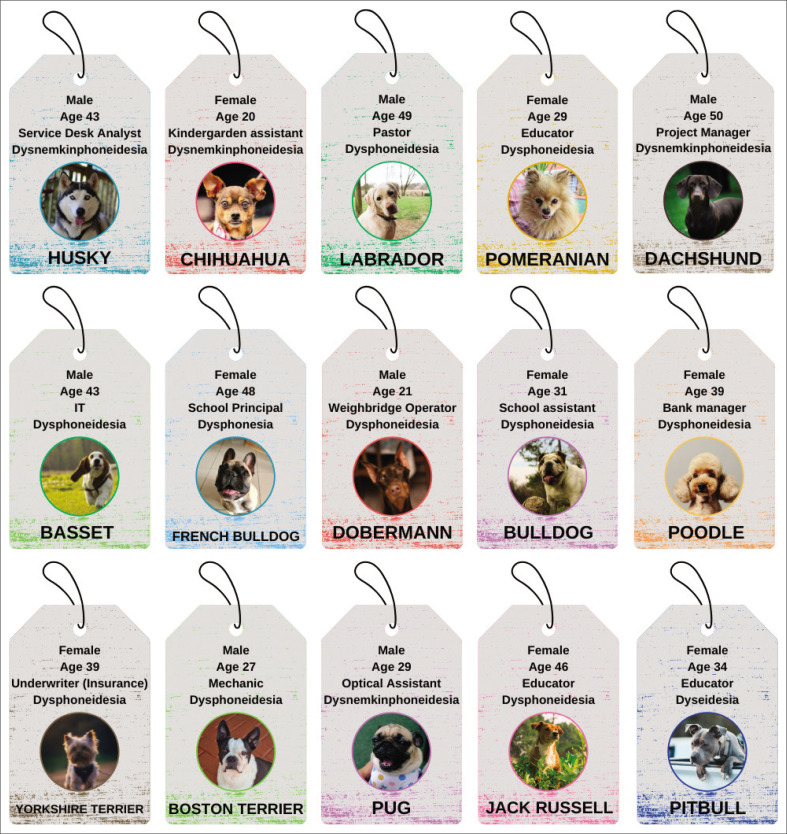
Pseudonyms and characteristics of employees.

**FIGURE 2:**
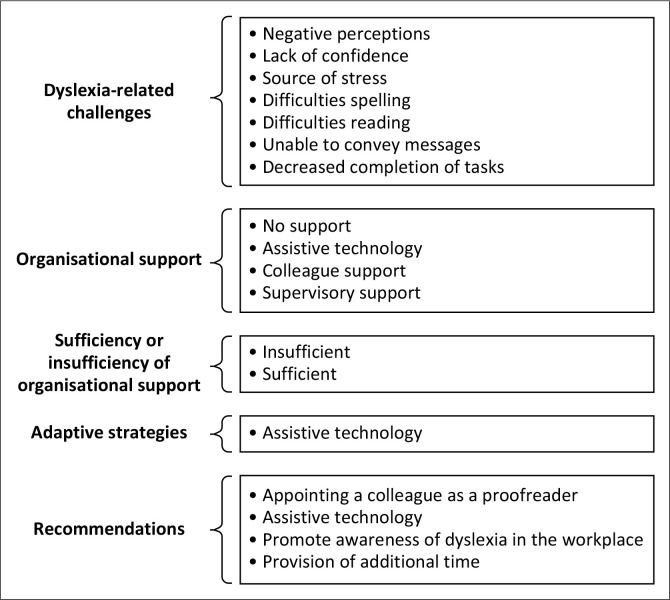
Overview of themes – Participant data.

This section presents the comprehensive findings of this research study. Five categories were identified: Dyslexia-related challenges; organisational support; sufficiency of organisational support; adaptive strategies; and recommendations. Figure 1 outlines the characteristics and pseudonyms used for the EWD who participated in this research study. Figure 2 provides an overview of the categories and themes extracted from the collected data.

### Theme 1: Dyslexia-related challenges

#### Negative perceptions

Most of the employees mentioned that a negative perception surrounds a dyslexia diagnosis. They further elaborated by stating that people often believe dyslexia is associated with stupidity, being unintelligent or incompetent:

‘I am trying to think, I know that especially in education, it is quite difficult when you have dyslexia because everyone thinks you are a paw-paw.’ (Pomeranian)

‘I think my biggest concern about dyslexia is that people think we are stupid. I wish someone would put an advertisement on TV at eight o’clock in the evening so that the whole world could see we are not stupid.’ (Jack Russell)

‘Dyslexia does not equal stupidity.’ (Labrador)

#### Lack of confidence

Some employees indicated that they often doubt themselves, thus causing them to believe that they have made a mistake either in the written work produced or in comprehending what was read. In addition, some of them have mentioned that they feel ashamed because they make spelling mistakes, which is not supposed to be like that:

‘So that has made it difficult because I now have to generate something and give it to them, and I am not confident with my language use.’ (Basset)

‘I get so frustrated at times that I doubt myself many times.’ (French Bulldog)

‘I cannot spell well, so I feel terribly embarrassed because I am actually in a professional profession.’ (Jack Russell)

#### Source of stress

Multiple employees indicated that being dyslexic is a source of stress for them. They often experience high levels of stress or anxiety when confronted with strenuous deadlines or when they, for example, have to read or write in the presence of others:

‘The other issue I have is reading, a big problem for me because I am a teacher, and I often have to read aloud. But when I read to the children, I become anxious, affecting me as well. It creates much tension.’ (Jack Russell)

‘Do not ask me to do it for you in a week because I will not be able to, and the stress you put on me by asking to do it in a week will mean I cannot get anything done.’ (Husky)

‘The stress one experiences because sometimes the workload is hefty, so you rush to finish everything on time.’ (Yorkshire Terrier)

#### Difficulties with spelling

Various employees indicated that spelling and reading are among the most significant challenges they experience daily. They further elaborated by stating that they prefer working on a computer because it is easier to ensure that your spelling is correct. Upon probing these EWD, they explained that this is because of them having spellcheckers on their computer, which assists them with their spelling, thus ensuring that the words are spelt correctly:

‘I have to type the word “there”, and I am forty-three and do not know which “there” is which “there”, and I come across “which”, and I do not know which “which” is which “which”.’ (Basset)

‘Do not ask me to write you a report, if they want something written, I would say wait a little bit. I grab my laptop; it is embarrassing if you cannot spell.’ (Dachshund)

‘Okay, I think my biggest challenge is easy, well, it is spelling.’ (Jack Russell)

#### Difficulties with reading

Many employees indicated that their most significant struggle is reading, including the speed at which they read and completely comprehending what is being read:

‘Reading is exhausting.’ (Pug)

‘My reading ability is slower.’ (Poodle)

‘So, for me, it is a challenge, the speed at which I read.’ (Basset)

‘I can recall how sometimes I have read something over six hundred and twenty times, and I still ask, “What the hell does this say?”.’ (Pitbull)

#### Unable to convey messages

Some employees indicated they needed help to convey the correct information or message in written form. Frequently, it makes sense in their head, but as soon as someone else reads it, it either does not make sense or the context is incorrect:

‘I think the challenge is to write it down correctly so that everyone can understand.’ (Yorkshire Terrier)

‘And again, in the workplace itself, you receive email after email, and you have to respond, but sometimes you think, “Okay, I will give this answer”, and then it is completely wrong because it has nothing to do with the question.’ (Pitbull)

‘The spellcheck program indicates that I spelt the word correctly, but it is the context that’s wrong.’ (Pug)

#### Decreased completion of tasks

Several employees indicated that because of the struggles of being dyslexic, it takes them much longer to complete specific tasks. They also mentioned that it takes them longer because they constantly double-check their work:

‘Of course, it takes me much longer than someone without dyslexia, to do the same work.’ (French Bulldog)

‘Okay, this is a bit more difficult for us than for an ordinary person because now you have to double-check everything.’ (Dobermann)

‘I have conditioned myself to read things two or three times just to make sure I read correctly the first time or the second time.’ (Poodle)

‘Pressure for time deadlines is always an issue because I always need more time than other people, as I cannot get through it because it is so much harder work for me.’ (Jack Russell)

### Theme 2: Organisational support

The research investigated organisational support for EWD. Employees with dyslexia reported on whether they received support and, if so, what kind. This data yielded the following themes:

#### No support from the organisation

Most employees indicated that they do not receive any support from their organisation. Some of them further explained that they would purchase it themselves if they needed support regarding their daily work life. Employees with dyslexia further explained that they would frequently purchase laptops with specific software such as spell checker or text-to-speech and speech-to-text programmes to assist them:

‘No, there is not any support.’ (Boston Terrier)

‘You know, I asked, and there is not, not for dyslexia.’ (Husky)

‘No, not at all, there is nothing like that.’ (Chihuahua)

‘No, not at all, I buy my own things like Grammarly, I buy it myself.’ (Basset)

#### Assistive technology

Some employees explained that the support they receive is in the form of assistive technology. Upon further analysis, it was found that this assistive technology consisted mainly of laptops or text-to-speech software:

‘They gave me a computer.’ (Dachshund)

‘There is an option where you can get audio on the documents, where they read it for you.’ (Poodle)

#### Colleague support

Multiple employees indicated that they primarily receive support from their colleagues in the workplace. Various examples of this type of colleague support were identified, including being able to ask for assistance when required, colleagues proofreading or double-checking work and, lastly, reading written work out loud:

‘I have the confidence now to tell him, “Here is what I am going to send for everyone to proofread, please check it for me.”’ (Basset)

‘If I stumble or if there is something I do not understand, then I can ask them, and they help me.’ (Bulldog)

‘There is always someone willing to just check for me whether everything is correct.’ (Yorkshire Terrier)

#### Supervisory support

Some employees suggested that their supervisors support them. Many employees indicated that their supervisors give them additional time or assist them by correcting or pointing out mistakes. Furthermore, some employees explained that when their supervisor understood what a dyslexic diagnosis entails, they felt confident enough to ask for assistance if and when required, regardless of the context:

‘They were very generous with additional time.’ (Labrador)

‘Yes, they always give me extra time.’ (Dachshund)

‘If I am struggling with something, I can just buzz her, or I just walk over to her office.’ (Pug)

‘Just that empathy is already enough for me.’ (Pitbull)

### Theme 3: Sufficiency or insufficiency of organisational support

Employee perceptions of organisational support varied. While some felt it was sufficient, others expressed concerns about its adequacy.

#### Insufficient

Various employees mentioned that they perceive the support to be insufficient. They further explained that they believe more can be done to support them. Some employees noted that they would like to see more support offered to them. However, they do not want to come across as selfish, or they might feel uncomfortable asking for additional support:

‘No, it is not sufficient. I feel they can do more to help us.’ (Husky)

‘I would want more, but not in a selfish way.’ (Pomeranian)

‘No, I think they can do more. The thing is, they help many other people who are in a wheelchair or something but do not use it.’ (Boston Terrier)

#### Sufficient

Several employees identified the support they received as sufficient. However, some employees explained that they feel it is their responsibility to ensure they have the necessary tools to succeed. Others also mentioned that although there is no support in the sense of, for example, assistive technology, their employer understanding their condition and having empathy towards them was sufficient:

‘I think it is adequate support.’ (Labrador)

‘More than adequate, yes.’ (Dachshund)

‘The assistance I receive from the employer is good enough for me because I am not afraid to ask.’ (Poodle)

‘I would say, in my opinion, it is, yes. So, my feeling is I do not want to burden the work with the fact that I read slowly or that I cannot spell.’ (Basset)

### Theme 4: Adaptive strategies

While not directly addressed in the interview questions, a recurring theme emerged: EWD independently adopted various strategies to improve their work experience. These strategies included acquiring assistive technology such as laptops, recording devices and reading pens (c-pens). Software programmes such as spell checkers, speech-to-text and text-to-speech tools, Grammarly and predictive text were also mentioned:

‘And by God’s grace, the voice-to-text technology has become so many leaps and bounds forward that even on this telephone that I am talking to you on now, I can press the microphone, and I can dictate into text for the phone, so I do not have to send a voice note when I have a long-winded message to send. That is the most wonderful thing available.’ (Labrador)

‘You find coping mechanisms, for example, with my spelling, I have three spell checkers that I use.’ (Husky)

‘I buy my own, like Grammarly, I buy it myself, and I use it.’ (Basset)

### Theme 5: Recommendations

Employees provided recommendations for improvements to identify preferred support structures. These suggestions aimed to enhance both effectiveness and efficiency in the workplace. The following themes emerged from the data:

#### Appointing a colleague as a proofreader

Several employees mentioned that they have the ability and skill to do the work. However, spelling is still a problem and needs improvement. They further explained that if they had someone within the organisation who could act as a language facilitator or proofread their work, it would be a great help:

‘You know, almost a bit like a language facilitator because I can do the work.’ (Jack Russell)

‘If I could have had someone, maybe, to read through my work again.’ (French Bulldog)

#### Assistive technology

Multiple employees mentioned that implementing assistive technology would make them more effective and efficient. The assistive technology mentioned consisted either of software (e.g., Text-to-speech programmes, speech-to-text programmes, Grammarly and Dragon NaturallySpeaking) or hardware (e.g., A reading pen or a recording device):

‘You know, if we are talking about aids, then I would tell you something simple like a Grammarly license.’ (Husky)

‘You know my biggest thing is speech-to-text technology that works across various platforms.’ (Labrador)

‘Like a pen or a thing that can read it for you.’ (Pug)

‘When I can rather record a meeting on an audio recording and then play it back for myself.’ (Pitbull)

#### Promote dyslexia awareness in the workplace

Employees with dyslexia emphasised a need for greater awareness within organisations. Employees with dyslexia believed that increased understanding among employers and colleagues would unlock their full potential. A recurring theme was the misconception that dyslexia is solely a childhood condition, further underlining the importance of awareness initiatives:

‘Because I have long thought that people are not good enough; that is why we work so hard in everything we do. So, I think employers should understand that, too. I think they would be amazed by what these people can mean to them.’ (French Bulldog)

‘You go to university, and you still struggle with your stuff, and in the workplace too. So, I think the problem is people think dyslexia only goes up until school.’ (Pug)

‘I lived under the illusion of what dyslexia means, but I have it and I did not realise, so definitely awareness.’ (Husky)

#### Provision of additional time

Most employees mentioned that merely being presented with additional time to complete tasks would greatly assist. Employees explained that they want additional time as it takes them longer to read and spell correctly. It was mentioned that additional time is especially required when the employees have to produce written work or complete paperwork:

‘Basically, to give me a bit more time on certain things, especially when I have paperwork and things to write out.’ (Dobermann)

‘Like they can give me their extra time.’ (Chihuahua)

‘Because it takes me a long time to read and spell, I think it would help me a lot if I could get more time to do my work.’ (Boston Terrier)

## Discussion

The general objective of this research study was to explore the organisational support provided to EWD within South African organisations. Although not part of the initial objectives of the study, ‘challenges related to dyslexia’ emerged as an incidental finding and will be discussed as a theme. This theme provides valuable insights into the perspectives of the dyslexic workforce within South Africa. Hence, this information was asked during the interview to obtain a clearer picture of each participant’s unique background and experience.

### Challenges related to dyslexia

Employees with dyslexia frequently reported experiencing a notable decrease in their speed when completing tasks, primarily because of the necessity for double-checking. While essential for ensuring accuracy, the meticulous double-checking had a crucial impact on their overall productivity. A study conducted by Greaney (2018) found that dyslexic individuals, because of the awareness of their condition, may naturally adopt a habit of double-checking, thus placing a strong emphasis on accuracy. Greaney (2018) further stated that non-dyslexic individuals might be more susceptible to lower vigilance levels than their dyslexic peers. Employees with dyslexia may experience difficulties with reading fluency and comprehension, leading to increased time and effort required for tasks involving written materials. This can hinder their ability to absorb information from reports, documents, and other workplace essentials. Our research supports this, suggesting that individuals with learning disabilities, such as dyslexia, often require additional time to accommodate the need for decoding words, which may not come naturally to them (Munzer, Hussain & Soares 2020; Nugent & Scott-Parker 2022).

Spelling challenges related to a daily hurdle for a considerable number of EWD. Many EWD found comfort in working on computers equipped with spell checkers, which ultimately assisted in ensuring correct and accurate spelling. This preference for technology highlights assistive technology’s role in mitigating the challenges faced by EWD. Assistive technology is a cornerstone in the functional capabilities of individuals dealing with operational challenges, thus enabling those individuals to actively participate and experience inclusion in all facets of life (World Health Organization 2022). Another significant challenge identified was expressing ideas or messages accurately in written format, as the intended message might not be conveyed correctly.

Self-doubt and shame periodically surfaced among EWD, particularly in response to spelling mistakes and perceived shortcomings in their work. These feelings of inadequacy not only affected their self-esteem but also impacted their job performance. A study conducted by Wissell et al. (2022a) found that EWD frequently struggle to build relationships because of their feelings of shame associated with having dyslexia. Furthermore, EWD even mentioned that they often experience a sense of imposter syndrome when they excel in their work (Wissell et al. 2022a). Many EWD in this research study also expressed frustration regarding the negative perceptions and stereotypes surrounding dyslexia. They often encountered misconceptions that linked dyslexia to incompetence or a lack of intelligence, whereas quite the opposite is true. One of the primary challenges dyslexic individuals face is the social stigma surrounding dyslexia, which can result in emotional difficulties such as anxiety, low self-esteem and frustration (MacCullagh 2014). Many EWD reported dyslexia and its challenges as a significant source of stress and anxiety, especially during public reading or writing tasks, which could negatively impact their well-being and performance.

### Types of organisational support provided to employees with dyslexia

A theme that surfaced from this research study falling within the dimensions of emotional support, as defined by Eisenberger and Stinglhamber (2011), was the presence of colleague support. Key aspects of this support include the ability to ask for help without judgement, colleagues proofreading or checking work and colleagues reading written work aloud. An open and supportive work environment empowers EWD to seek assistance, fostering well-being and potentially improving performance. Kirby and Gibbon (2018) support this finding by highlighting the importance of developing support networks within an organisation. Furthermore, they mention that to support EWD better, organisations should implement a comprehensive approach that combines the availability of peer support for ad hoc assistance with thorough dyslexia awareness training.

The study underscores the importance of assistive technology, falling within the dimension of instrumental support defined by Eisenberger and Stinglhamber (2011), in supporting EWD within the organisation. Two key aspects emerged within the theme of workplace accommodations: providing laptops and implementing text-to-speech software. Organisations offering personal laptops and text-to-speech tools empower EWD by improving their reading and comprehension abilities. The Dyslexia Association (2023) emphasises the value of assistive technology for EWD. Tools such as mind mapping software, text-to-speech and smartpens can significantly improve their daily work experience. By offering these resources, organisations can empower EWD and enhance their productivity. These assistive technology tools are also better known as augmentative and alternative communication (ACC) (Beukelman & Light 2020). Augmentative and alternative communication refers to a collection of tools and strategies individuals use to address daily communication challenges such as speech and writing International Society of Augmentative and Alternative Communication [ISAAC] 2011). Different types of ACC exist, such as no-tech, low-tech and high-tech options. High-tech options include using apps on tablets or iPads to communicate and speech-generating devices (American Speech-Language-Hearing Association 2025; Beukelman & Light 2020). In the context of ACC, strategic competence also refers to the ability of individuals to utilise various strategies to effectively overcome the challenges that they are experiencing when communicating (Beukelman & Light 2020). In a study conducted by McNaughton, Light and Gulla (2003), it was found that when employees use ACC within the workplace, it may lead to increased loyalty, performance talents and skills.

This study highlights a lack of support for EWD in some workplaces. The absence of accommodations can negatively impact performance, increase absenteeism and limit employee abilities (Prince 2017). Reinforcing this, Morris, Begel and Wiedermann (2015) found that a vast majority (94.1%) of EWD who disclosed their condition received no workplace accommodations. Wissell et al. (2022b) identified a lack of training among employers and managers in supporting EWD. Their research suggests that training programmes on dyslexia awareness could improve support and understanding for EWD, colleagues and the entire organisation. These initiatives could also reduce stigma and discrimination.

This study highlights the importance of supervisory support for EWD, encompassing both emotional and practical aspects. Supervisors can positively impact performance by granting extra time, providing constructive feedback and fostering a safe environment through dyslexia awareness. Training for line managers on supporting EWD and raising dyslexia awareness within the organisation is crucial (Kirby & Gibbon 2018; Wissell et al. 2022b).

### Effectivenessof organisational support provided to employees with dyslexia

This research study revealed various perspectives among EWD regarding the sufficiency or organisational support received. Some EWD indicated that they are satisfied with the support received and that it is sufficient, emphasising their willingness to ask for assistance when required. These EWD conveyed that they perceive the support as sufficient because they feel comfortable and safe enough to seek assistance, such as help with reading, whenever needed. They noted that their colleagues and employers consistently are willing to provide that assistance. Employees with dyslexia further mentioned that the support was sufficient, enabling them to overcome challenges effectively. However, many of them believe the existing support fell short of their needs. They highlighted the fact that the organisation could take additional measures to comprehensively accommodate EWD. Within the subsequent objective discussion, EWD explained why the existing support may have fallen short of their needs. In addition, they also made recommendations and suggestions on how organisations can address their specific and unique needs.

Interestingly, some EWD drew attention to the discrepancy between the support provided for physical disabilities (e.g., wheelchair accessibility) and that for dyslexia, a blind disability. These discrepancies emphasise the need for organisations to recognise and address the invisible nature of dyslexia-related challenges. Notably, the *Employment Equity Act 55 of 1998* in South Africa states that all employers and organisations must implement and provide reasonable accommodations for the South African workforce with disability regardless of the type of disability an employee presents (Department of Labour 2015). According to the British Dyslexia Association (n.d.), when implementing reasonable accommodations, organisations must assess the nature and severity of the individual’s dyslexia, consider the job role and its prerequisites, which can be performed through a Workplace Needs Assessment, evaluate the impact of the working environment and address the requirements for associated training.

The law, therefore, mandates the implementation of reasonable accommodations for individuals with disabilities, thus emphasising organisational compliance and adherence to these requirements.

### Adaptive strategies

Although no specific question was posed to the EWD during the interviews, an interesting finding revolved around the adaptive strategies Employees with dyslexia independently developed or implemented to navigate their work environment and daily work-related tasks more effectively. The theme of assistive technology emerged. To clarify, the earlier mentioned assistive technology pertains to technology provided by the organisation. In contrast, adaptive strategies involving assistive technology refer to tools and solutions that EWD have personally acquired or implemented to enhance their daily work life, distinct from offerings provided by the organisation. Employees with dyslexia detailed their use of various hardware and software tools to mitigate the impact of dyslexia on their tasks. Hardware devices such as laptops, recording devices and C-pens emerged as valuable tools for note-taking and information retention.

Software-based adaptive strategies played a crucial role as well. Spell checkers, speech-to-text and text-to-speech programmes were highlighted for enhancing written communication. Employees with dyslexia mentioned using tools such as Grammarly and predictive text programmes to ensure accuracy and clarity in their written work. Grammarly provides robust real-time communication support, harnessing AI to assist in composing and revising content while enhancing typed work by refining grammar, tone and clarity (Grammarly 2023). Moreover, it incorporates predictive text, aiding users as they type (Grammarly 2023) – a tool that can be extremely useful for EWD in their daily work lives.

### Recommendations by employees with dyslexia to enhance organisational support

Employees with dyslexia might often struggle with written communication, including spelling and grammar errors and conveying intent accurately, which can be particularly challenging in a professional setting. For this reason, EWD suggested assigning a colleague as a proofreader. A supportive colleague can ensure error-free work while fostering collaboration. Research highlights the importance of empathy and collaboration skills in managers for dyslexic employee success (Wissell et al. 2022b). It was therefore recommended that to create a supportive work environment, managers be trained on dyslexia and how to effectively support a dyslexic employee. A truly inclusive work environment can be created when managers are supportive and receive training on dyslexia (Cullen, Darby & Rahmani 2019).

Employees with dyslexia stressed the importance of assistive technology like text-to-speech and spell checkers within the workplace. Although expensive to invest in, these tools can boost productivity. They further recommended that dyslexia awareness be promoted in workplaces. This recommendation focuses on eliminating misconceptions and stigmas associated with dyslexia. Employees with dyslexia indicated that promoting dyslexia awareness will assist managers and colleagues in understanding their daily challenges better, and they can adapt their communication and expectations while providing adequate support. Research suggests a stigma surrounds dyslexia, hindering employment and open communication (Alexander-Passe 2015).

In this study, EWD requested extra time for tasks, acknowledging their slower reading and writing capabilities. This could level the playing field and ensure they can showcase their skills. Research confirms dyslexia’s impact on work performance (Bartlett et al. 2010; Beetham & Okhai 2017; De Beer et al. 2014; Nalavany, Logan & Carawan 2018) and highlights the need for support to ensure dyslexic employee success.

To summarise, dyslexia can significantly impact people’s lives, especially professionally. However, with the appropriate support and accommodations, the challenges faced by EWD can be mitigated, helping them to achieve success in the organisation. South Africa’s *Employment Equity Act 55 of 1998* outlines the necessity and employer responsibility to implement and provide reasonable accommodations to the South African workforce where required (Department of Labour 2015).

### Practical implications

This research highlights the need for dyslexia awareness training in South African workplaces. Open communication about dyslexia is crucial, and organisations should prioritise personalised support plans and assistive technologies to empower EWD.

### Strengths and limitations

This qualitative study allowed EWD to share their thoughts and experiences, which is not always possible with quantitative data. This study provided a voice to EWD, allowing their experiences and viewpoints to be heard. The researcher could probe them for answers during the interviews, further enhancing the data’s comprehensiveness.

Limitations of this study included the limited language diversity. Most of the participants were Afrikaans-speaking (86.7%), and their experiences may primarily reflect those of Afrikaans-speaking EWD, thereby limiting the transferability to other language groups. Qualitative research is often viewed as subjective because of the personal interpretations of the researcher when it comes to data collection and analysis. However, the researcher ensured that she remained unbiased and objective when analysing the findings of this study. Also, the researcher and co-coder utilised the same data analysis technique. The co-coder also assisted the researcher in ensuring that the interpretation of the results was unbiased and objective.

### Recommendations

Future research recommendations include a larger, more diverse sample across South Africa. Quantitative surveys with text-to-speech options to reach a broader audience might be considered. Conducting longitudinal studies is imperative to ensure the sustainability and effectiveness of support and accommodation strategies for EWD. These studies can track the long-term impacts of these strategies on employees’ careers, job satisfaction and overall well-being. Moreover, any potential barriers or unintended consequences arising from these accommodations can be identified and addressed to refine support mechanisms further.

A safe and inclusive workplace culture should be embedded in the organisation’s values and practices. This entails promoting diversity and inclusion at all levels, from leadership to entry-level positions. By fostering a culture that values all employees’ unique perspectives and talents, organisations can create an environment where everyone, including those with dyslexia, can thrive and contribute to their fullest potential. This cultural shift towards inclusivity should be an ongoing commitment, reflecting the organisation’s dedication to supporting all its members. Organisations can also consider developing personalised support plans for EWD. Investing in dyslexia training programmes for all employees is also suggested. By acknowledging the prevalence of dyslexia and proactively creating a supportive environment, organisations can benefit from the talents of EWD.

Furthermore, organisations should establish mechanisms for periodically evaluating the effectiveness of support strategies provided. Feedback from EWD should be actively sought and used to refine and optimise the tools provided. This approach ensures that the support offered is beneficial and aligned with the evolving needs of these employees.

## Conclusion

In conclusion, EWD yielded valuable insights into the multifaceted challenges and opportunities surrounding dyslexia in the workplace. This research has delved into several critical dimensions of this subject, shedding light on various aspects of organisational support, the experiences of EWD and their recommendations for improvement. Dyslexia, as a lifelong condition, presents significant challenges in reading, writing and other daily tasks encompassing literacy skills. By understanding dyslexia’s challenges, organisations can create a more inclusive environment where EWD can thrive alongside their peers. This study underscores the importance of understanding the intricacies of dyslexia and how it impacts individuals’ professional lives. Employees with dyslexia, like all individuals, deserve an equal opportunity to thrive and succeed professionally, and these findings provide a path forward to achieving this goal.
